# Painful subcutaneous edema is associated with early age at disease onset in Immunoglobulin A vasculitis patients: A multicenter study

**DOI:** 10.1016/j.clinsp.2025.100857

**Published:** 2026-01-09

**Authors:** Lia V. Steuer, Clara R. Doria, Matheus S. França, Paula S. Marra, Sebastian D. Cordoba, Luisa F.C. Forero, Ricardo N. Machado, Sylvia C.L. Farhat, Gleice Clemente, Vitória Curi, Claudio A. Len, Luciana M. Carvalho, Francisco H.R. Gomes, Virginia P.L. Ferriani, Rozana G. de Almeida, Flavio R. Sztajnbok, Lucia M.A. Campos, Adriana M. Elias, Verena A. Balbi, Nadia E. Aikawa, Beatriz O.L. Carneiro, Magda Carneiro-Sampaio, Katia T. Kozu, Clovis A.A. Silva

**Affiliations:** a Pediatric Rheumatology Unit, Instituto da Criança e do Adolescente, Hospital das Clínicas da Faculdade de Medicina da Universidade de São Paulo, São Paulo, SP, Brazil; b Pediatric Rheumatology Unit, Universidade Federal de São Paulo, São Paulo, SP, Brazil; c Pediatric Rheumatology Unit, Faculdade de Medicina de Ribeirão Preto, Universidade de São Paulo, Ribeirao Preto, SP, Brazil; d Rheumatology Division, Instituto de Puericultura e Pediatria Martagão Gesteira - Universidade Federal do Rio de Janeiro, Rio de Janeiro, RJ, Brazil

**Keywords:** Immunoglobulin a vasculitis, Henoch-schönlein purpura, Edema, Children

## Abstract

**Objective:**

To evaluate the risk factors associated with the presence of Painful Subcutaneous Edema (PSE) in children and adolescents with Immunoglobulin A Vasculitis (IgAV).

**Methods:**

A multicenter study evaluated 686 patients (≤ 18 years-old) at first 3-months after diagnosis. IgAV patients with PSE were compared to those without PSE.

**Results:**

PSE was found in 219/686 (31.9 %). The sites were lower limbs 192/215 (89.3 %) and upper limbs 85/215 (39.5 %). Persistent PSE (≥ 6-weeks of duration) was found in 4/215 (2 %), and recurrent PSE was found in 7/217 (3 %). The median age at diagnosis was significantly lower in PSE patients compared to those without [5.0 (3.4) vs. 6.3 (4.3) years, *p* = 0.001]. Increased CRP was significantly higher in IgAV with PSE compared to without PSE (52.6 % vs. 41.1 %, *p* = 0.03), likewise thrombocytosis (> 400.000 mm^3^) (43.8 % vs. 35.1 %, *p* = 0.04). Although associated with higher inflammatory markers, PSE was not linked to more severe outcomes. Logistic regression demonstrated that age at diagnosis was inversely associated with PSE (OR = 0.986; 95 % CI 0.981‒0.992; *p* < 0.001).

**Conclusion:**

PSE occurred in approximately one-third-of IgAV patients at disease onset and was diagnosed predominantly at an early age, with a more inflammatory presentation at onset. However, in spite of the higher levels of inflammatory markers, PSE was not linked to more severe outcomes.

## Highlights

• PSE occurred in approximately one-third of IgAV patients at disease onset.
• IgAV painful subcutaneous edema was mainly located on the lower and upper limbs.
• IgAV painful subcutaneous edema was identified predominantly at an early age.

## Introduction

Immunoglobulin A (IgA) Vasculitis (IgAV), formerly Henoch-Schönlein purpura, is the most common systemic vasculitis in childhood, characterized by small vessel involvement and IgA deposit in the affected vessels.^1^^,^^2^ The clinical spectrum of skin manifestations includes purpura, petechiae, hemorrhagic vesicle, bullae, blister, ulcerations, necrosis, nodule and Painful Subcutaneous Edema (PSE).^2^, ^3^, ^4^, ^5^

PSE in children and adolescents with IgAV is mostly characterized by a non-pitting inflammatory edema associated with localized tenderness upon palpation,^6^^,^^7^ and has been reported from 4.5 % to 63.2 % of IgAV patients.^3^^,^^8^, ^9^, ^10^, ^11^, ^12^, ^13^, ^14^

The wide variability in PSE prevalence reported in the literature (4.5 %‒63.2 %) can be attributed to substantial differences in study populations, sample sizes, and methodologies. Earlier reports were based on small cohorts of fewer than 50 patients and focused mainly on young children,^3^^,^^8^ whereas more recent studies included larger series participants and included a broader pediatric spectrum extending into adolescence.^9^, ^10^, ^11^, ^12^, ^13^, ^14^

PSE generally occurs at disease onset, mainly localized in the lower and upper limbs.^6^^,^^9^^,^^13^^,^^15^ Involvement of other anatomical regions, such as the face, lumbar area, scalp and periorbital region has also been reported.^3^^,^^6^^,^^7^^,^^13^^,^^16^, ^17^, ^18^, ^19^

Of note, this feature has been described mainly in case reports and case series.^7^^,^^15^^,^^17^, ^18^, ^19^, ^20^ A previous report showed that PSE in IgAV patients occurred predominantly in younger patients.^3^ However, to our knowledge, no studies have evaluated the risk factors associated with the presence of PSE in a large IgAV population.

Therefore, the objective of the present study is to evaluate initial factors of IgAV associated with PSE and its outcomes in children and adolescents in a large multicenter study.

## Methods

A multicenter study involving four university referral centers in Brazil enrolled 705 children and adolescents (≤18-years-old) with IgAV within the first 3-months after diagnosis. Of these, 19 patients were excluded because they did not fulfill the European League Against Rheumatism/Paediatric Rheumatology International Trials Organisation/Paediatric Rheumatology European Society (EULAR/PRINTO/PRES) classification criteria for IgAV^21^ (*n* = 14), incomplete medical records (*n* = 3), and had age > 18-years at diagnosis (*n* = 2). The remaining 686 IgAV patients were included in the present study. None of them had Finkelstein-Seidlmayer vasculitis, also named acute hemorrhagic edema of young children or infantile IgAV.^21^

An online investigator meeting was held before data collection to refine and standardize the study protocol, which included detailed definitions for demographic, clinical, therapeutic, and outcome variables. Each participating center had a designated coordinator responsible for supervising local data entry. Investigators and research staff who completed the REDCap database were specifically trained and certified to ensure uniformity and reliability of data collection. All doubts or inconsistencies were discussed collectively among investigators from all centers to achieve consensus and improve data standardization. Data quality control involved three independent review rounds to identify and correct erroneously entered or missing information. Outlier and extreme values were systematically verified against the original medical records and, when necessary, confirmed or corrected. This multi-step validation process aimed to minimize discrepancies across the four centers and ensure the final dataset's accuracy and completeness. Data collection occurred between December 2023 and April 2025. Patients were diagnosed between 1981 and 2023 and followed from 1981 to 2025.

The charts were systematically and retrospectively assessed for demographic data, initial clinical manifestations, laboratory exams, treatments, and outcomes collected at baseline and during follow-up at 1-, 5-, 10-, and 15-years after diagnosis. The study received Ethical Committee Approval from all participating centers in 2023. For this study, the authors used only retrospective data; therefore, informed consent was not required.

Patients were grouped according to the presence or absence of PSE within the first three months. Affected locations of PSE included: lower limbs, upper limbs, face, scalp, back, and chest. The duration of PSE (in days) was also recorded. Persistent PSE was defined as lasting for ≥ 6-weeks, while recurrent PSE was considered a new episode following complete resolution.^6^^,^^23^

Demographic variables included age at diagnosis, sex and interval between symptom onset and diagnosis. Body Mass Index (BMI) was calculated as weight in kilograms divided by height in meters squared (kg/m^2^). Potential triggers such as recent streptococcal infection and the need for penicillin prophylaxis were evaluated.

Skin involvement was a mandatory inclusion criterion and was defined as the presence of purpura and/or petechiae. Recurrent lesions were considered new occurrences after complete resolution, while persistent lesions were identified as those lasting for ≥ 6 weeks.^23^

Arthritis was characterized as joint edema or pain with limited range of motion, whereas arthralgia referred to joint pain without swelling or movement restriction.^21^ Persistent arthritis was identified as this musculoskeletal involvement lasting ≥ 6-weeks, whereas recurrent arthritis referred to a new episode following full recovery.^23^

Severe abdominal pain was characterized by at least one of the following signs or symptoms: peritoneal irritation, intussusception, or gastrointestinal bleeding. Recurrence of abdominal pain was considered a new episode after resolution^24^ and persistence referred to symptoms lasting ≥ 6-weeks.^23^ Associations with food allergies and the use of abdominal ultrasound for confirmation of abdominal complications were also analyzed.

Renal involvement was assessed by the presence of hematuria (> 5 red blood cells per high-power field or red cell casts in urine), proteinuria > 0.1 g/m^2^/day, leukocyturia and cylindruria. Arterial hypertension was defined as systolic and/or diastolic values ≥ 95th percentile for sex, age, and height on at least three separate occasions.^25^^,^^26^ Nephrotic syndrome was defined as edema, serum albumin < 2.5 g/dL, and nephrotic proteinuria (24h proteinuria > 40 mg/m^2^/hour [1000 mg/m^2^/day] or protein/creatinine ratio > 2.0) .^21^^,^^24^ Acute Kidney Injury (AKI) was identified based on oliguria lasting more than 6-hours, a rapid rise in serum creatinine exceeding 0.3 mg/dL within a 48-hour period, or an increase greater than 50 % over the course of one week.^27^ Chronic Kidney Disease (CKD) was characterized by the presence of structural or functional renal alterations persisting for at least three months, or by a sustained reduction in Glomerular Filtration Rate (GFR) below 60 mL/min/1.73 m^2^ over the same period.^28^^,^^29^ Renal replacement therapies (hemodialysis, peritoneal dialysis and/or transplantation) were also recorded. Renal biopsy indications in IgAV cases included severe nephrotic syndrome, AKI or CKD, or persistent abnormalities (e.g., hematuria or proteinuria > 0.1 g/m^2^/day for > 3-months) .^24^ Biopsies were classified in six different subtypes using criteria from the International Study of Kidney Disease in Children (ISKDC) .^30^

Scrotal involvement was defined as orchitis/orchiepididymitis by the concomitant presence of edema and tenderness on physical examination and/or abnormalities on testicular Doppler ultrasound.^31^^,^^32^ Neuropsychiatric involvement was defined by the presence of any sign or symptom such as headache, seizures, loss of consciousness or localized signs. Ophthalmological manifestation was characterized by abnormal eye exam findings. Pulmonary manifestations were identified as the presence of cough, hemoptysis, dyspnea and tachypnea.^33^

Reference ranges for laboratory parameters were defined according to the standards provided by each participating center’s clinical laboratory. Serum IgA levels were measured using methods such as automated turbidimetric and nephelometric techniques. C-Reactive Protein (CRP) was evaluated by immunoassays or laser nephelometry and Erythrocyte Sedimentation Rate (ESR) by the Westergren method.

The IgAV treatment at the first three months after diagnosis were collected: glucocorticosteroids (prednisone or intravenous metilprednisolone pulsoterapy), antiproteinuric agents, immunosuppressive agents use (methotrexate, azathioprine cyclosporine, mycophenolate and/or cyclophosphamide), and intravenous immunoglobulin.

## Statistical analysis

Data were illustrated as median (range) or mean ± Standard Deviation (SD) for continuous variables, and as frequency (percentage) for categorical variables. Comparisons between IgAV patients with and without PSE were performed using the Mann-Whitney *U* test or Student's *t*-test for continuous variables, and Fisher’s exact test for categorical variables. Logistic regression analysis models were done using PSE as a dependent variable and variables that presented a statistical significance level of *p* < 0.2 in the univariate analyses as independent variables. Statistical significance was set at *p* < 0.05. Statistical significance was set at *p* < 0.05.

## Results

PSE was found in 219/686 (31.9 %) of IgAV patients during the first 3-months after diagnosis. The median duration of PSE was 7-days (1‒60). The most important located sites were lower limbs 192/215 (89.3 %), upper limbs 85/215 (39.5 %), face 29/215 (13.4 %), scalp 5/215 (2.3 %), back 3/215 (1.4 %) and chest 2/215 (0.9 %). Multiple locations of PSE occurred in 81/215 (37.6 %). Persistent PSE (≥ 6-weeks of duration) was found in 4/215 (2 %) and recurrent PSE was found in 7/217 (3 %). At disease onset, only 2/35 (5.7 %) patients with nephrotic syndrome had concomitant PSE with edema duration of 5-days and 17-days, respectively.

PSE recurrence was a rare event during follow-up. It was observed in 3/561 (0.5 %) of IgAV patients at the first year after diagnosis, 1/406 (0.2 %) at 5-years, 1/163 (0.6 %) at 10-years, and no case was identified at 15-years of follow-up. PSE recurrences were concomitant to new IgAV episodes. Only one IgAV patient presented PSE only after 5-years of diagnosis and without previous occurrence of this manifestation.

Table 1 shows demographic data and clinical manifestations in 686 IgAV patients with and without PSE at the first 3-months after diagnosis. The median age at IgAV diagnosis was significantly lower in PSE patients compared to those without this manifestation (5.0 [3.4] vs. 6.3 [4.3] years, *p* = 0.001). The findings demonstrated a one-day higher duration of purpura and/or petechiae in the PSE group (15 ^18^ vs. 14 ^18^ days, *p* = 0.03) and a significantly higher frequency of petechiae between both groups (52.5 % vs. 41.3 %, *p* = 0.01). No differences were evidenced in the other demographic data and clinical manifestations in IgAV patients with and without PSE (Table 1).

**Table 1:** Demographic data and clinical manifestations in 686 IgA vasculitis patients with and without Painful Subcutaneous Edema (PSE) at diagnosis.

Variables at diagnosis, n ( %)	With PSE (*n* = 219)	Without PSE (*n* = 467)	p
Demographic data			
Age at diagnosis, years	5.0 (3.4)	6.3 (4.3)	0.001
Male sex	108 (49.3)	232 (49.7)	0.94
Days between age of onset and age at diagnosis	8 (16)	7.0 (16)	0.38
Body mass index, kg/m^2^	16.2 (3.3)	16.5 (3.0)	0.16
Triggers and penicillin prophylaxis			
Recent streptococcus infection	14/95 (14.7)	26/154 (16.9)	0.72
Penicillin prophylaxis use	14/218 (6.4)	25/462 (5.4)	0.59
Purpura and/or petechiae	219 (100)	466 (99.8)	1.00
Persistent	21/216 (9.7)	38/458 (8.3)	0.98
Recurrent	38/211 (18)	62/448 (13.8)	0.16
Duration, days	15 (18)	14 (18)	0.03
Petechiae	115/219 (52.5)	193/467(41.3)	0.01
Arthritis and/or arthralgia	174/218 (79.8)	384/467 (82.3)	0.46
Persistent	3/173 (1.7 %)	8/375 (2.1)	1.00
Recurrent	6/173 (3.5)	20/377 (5.3)	0.39
Duration, days	7.0 (7)	7.0 (10)	0.19
Gastrointestinal involvement			
Abdominal pain	136/219 (62.1)	283/467 (60.6)	0.74
Recurrent abdominal pain	17/136 (12.5)	35/276 (12.7)	1.00
Severe/ moderate abdominal pain	60/109 (55)	110/245 (44.9)	0.11
Abdominal pain duration, days	5.0 (9)	7.0 (11)	0.08
Association with food allergy	0 (0)	1/277 (0.4)	1.00
Gastrointestinal bleeding	23/136 (16.9)	55/279 (19.7)	0.51
Bowel intussusception	1/134 (0.7)	1/279 (0.4)	0.54
Signs of peritoneal irritation	8/136 (5.9)	13/276 (4.7)	0.82
Renal involvement	72/219 (32.9)	125/467 (26.8)	0.10
Arterial hypertension	13/167 (7.8)	25/355 (7.3)	0.86
Nephrotic syndrome	2/35 (5.7)	12/65 (18.5)	0.13
Acute kidney injury	10/72 (13.9)	22/120 (18.3)	0.55
Leukocyturia	35/72 (48.6)	42/117 (35.9)	0.09
Urinary casts	4/71 (5.6)	6/116 (5.2)	1.00
Hematuria	54/71 (75.0)	102/122 (83.6)	0.19
Proteinuria	37/71 (52.1)	68/122 (55.7)	0.66
Chronic kidney disease	0 (0)	0 (0)	1.00
Orchitis	17/219 (7.8)	40/467 (8.5)	0.84
Recurrent	0 (0)	1/40 (2.5)	1.00
Duration, days	5.0 (4)	4.0 (6)	0.64
Neuropsychiatric involvement	1/219 (0.5)	1/467 (0.2)	0.54
Pulmonary vasculitis	0 (0)	1/467 (0.2)	1.00
Ocular involvement	0 (0)	0 (0)	1.00

Results are presented as median (IQR) or n ( %).

Table 2 illustrates laboratory tests and treatment in 686 IgAV patients with and without PSE at the first 3-months after diagnosis. Increased CRP was significantly higher in IgAV with PSE compared to those without PSE (52.6 % vs. 41.1 %, *p* = 0.03), likewise, thrombocytosis (> 400.000 mm^3^) (43.8 % vs.35.1 %, *p* = 0.04). No differences were shown in the other laboratory tests and treatment in IgAV patients with and without PSE (Table 2).

**Table 2:** Laboratory findings and treatment in 686 IgA vasculitis patients with and without Painful Subcutaneous Edema (PSE) at diagnosis.

Variables at diagnosis, n ( %)	With PSE (*n* = 219)	Without PSE (*n* = 467)	p
**Laboratory abnormalities**			
Increased serum IgA (> 255 mg/dL)	23/66 (34.8)	20/92 (21.7)	0.07
Increased ESR	80/151 (53)	150/315 (47.6)	0.32
Increased CRP	80/152 (52.6)	111/270 (41.1)	0.03
Anemia (Hb < 10 g/dL)	9/219 (4.1)	8/467 (1.7)	0.68
Leucocytosis (> 10,000 mm^3^)	101/219 (46.1)	203/467 (43.5)	0.51
Thrombocytosis (> 400,000 mm^3^)	96/219 (43.8)	164/467 (35.1)	0.04
Increased ASO (> 200 UI/mL)	33/68 (48.5)	76/138 (55.1)	0.46
**Treatments**			
Prednisone	116/219 (53)	211/462 (45.7)	0.08
Intravenous metilprednisolone pulsoterapy	15/219 (6.8)	33/455 (7.3)	1.00
Prednisone dose, mg/kg/day	1.0 (1)	1.0 (1)	0.48
Prednisone duration, days	30.0 (50)	30.0 (53)	0.97
Antiproteinuric agents	11/219 (5.0)	20/460 (4.3)	0.70
Immunosuppressive agents	3/219 (1.4)	6/467 (1.3)	0.69
Intravenous immunoglobulin	1/218 (0.5)	2/458 (0.4)	1.00
Renal replacement therapy	0 (0)	0 (0)	1.00

Results are presented as median (IQR) or n ( %). ASO, Anti-Streptolysin O.

Further analysis comparing multi-site and single-site PSE involvement demonstrated similar frequencies of increased serum IgA (> 255 mg/dL) (*p* = 0.20), increased ESR (*p* = 0.30), increased CRP (*p* = 0.74), anemia (Hb < 10 g/dL) (*p* = 0.72), leukocytosis (> 10,000 mm^3^) (*p* = 0.78), thrombocytosis (> 400,000 mm^3^) (*p* = 0.57) and increased ASO (> 200 UI/mL) (*p* = 0.62) in both groups (Table 3).

**Table 3:** Laboratory findings in 215 IgA vasculitis patients with painful subcutaneous edema PSE with multiple locations versus to single-site involvement.

Variables at diagnosis, n ( %)	Multiple-site PSE (*n* = 81)	Single-site PSE (*n* = 134)	p
Laboratory abnormalities			
Increased serum IgA (> 255 mg/dL)	9/34 (25.5)	14/32 (43.8)	0.20
Increased ESR	40/62 (64.5)	39/86 (45.3)	0.30
Increased CRP	34/62 (54.8)	45/88 (51.1)	0.74
Anemia (Hb < 10 g/dL)	4/81 (4.9)	5/134 (3.7)	0.72
Leukocytosis (> 10,000/mm^3^)	36/81 (44.4)	63/134 (47.0)	0.78
Thrombocytosis (> 400,000 mm^3^)	33/81 (40.7)	61/134 (45.5)	0.57
Increased ASO (> 200 UI/mL)	13/30 (43.3)	18/36 (50.0)	0.62

Results are presented as n ( %), ASO = anti-streptolysin O.

Logistic regression analysis was performed, including five independent variables: age at diagnosis, presence of petechiae, recurrence of purpura/petechiae, duration in days of purpura/petechiae, and thrombocytosis. Age at diagnosis was the only variable inversely associated with PSE (OR = 0.986; 95 % CI 0.981‒0.992; *p* < 0.001).

## Discussion

This was the first multicenter study to demonstrate that PSE occurred in approximately one-third-of IgAV patients at disease onset, mainly located on lower and upper limbs, and identified predominantly at an early age.

The present study has strengths. The sample size was the largest that analyzed PSE with long-term assessment, and patients were followed up at the university and tertiary centers in a Latin American continental country. All IgAV patients were children and adolescents and fulfilled the validated classification criteria of EULAR/PRINTO/PRES for IgAV.^21^ Another relevant advantage of this study was the use of a standardized database to minimize bias, including a systematic evaluation of 15-years of follow-up.

More importantly, the authors excluded patients with acute hemorrhagic edema, since a recent systematic review showed that only one fourth of these patients presented IgA deposits documented in immunofluorescence of skin biopsy specimens, indicating distinct pathophysiologic mechanisms of this condition and IgAV.^22^ Another strength of the present study was the differentiation between PSE and non-painful edema of renal origin, which is one of the cardinal clinical features of nephrotic syndrome and can vary from mild periorbital puffiness to generalized edema (anasarca) .^34^ In addition, renal edema is persistent in IgAV-related renal disease, contrasting with short-duration observed in the only two PSE patients with nephrotic syndrome identified in this population (5- and 17-days, respectively). The authors extended previous observation demonstrating that age at diagnosis was the only variable inversely associated with PSE.^3^ Nussinovitch et al., 1998 reported that a subgroup of IgAV with edema was significantly younger compared to the cohort in general.^3^ However, these authors did not compare clinical manifestations, treatments and outcomes in IgAV patients with and without PSE.

In univariate analysis, the present study identified that PSE was linked to a small variation in purpura and/or petechiae duration. Although a statistically significant difference was observed between groups, this finding has limited clinical relevance; the one-day difference is unlikely to represent a meaningful impact on disease course or prognosis. In addition, although the present data showed that PSE was associated with higher inflammatory markers, these findings were not related to more severe outcomes, particularly renal involvement, severe abdominal pain, or treatment with aggressive immunosuppressive agents. Furthermore, persistent or recurrent PSE was rare in IgAV patients. When present, the swelling typically resolved over time without causing serious complications.

Previous studies reported that subcutaneous edema in IgAV ranged from 4.5 % to 63.2 %^3^, ^4^, ^5^, ^6^, ^7^, ^8^, ^9^, ^10^, ^11^, ^12^, ^13^, ^14^ mainly affecting lower and upper limbs,^9^^,^^13^^,^^14^^,^^35^ as also observed herein. Atypical locations of PSE in IgAV patients have also been documented, as also evidenced herein. Periorbital and scalp edema have been described in a minority of patients,^13^ likewise the lumbar region and face.^6^^,^^7^^,^^19^ Furthermore, multiple localizations of PSE occurred in more than one-third of our patients, indicating a systemic inflammatory mechanism of this skin manifestation.

While the retrospective design was a major weakness, investigator supervision at each center limited this methodological flaw. This supervision ensured a low incidence of missing data. Another limitation of this study was the presence of missing data regarding PSE location. This discrepancy reflected incomplete documentation in the original medical records, it is unlikely to have compromised the study’s main findings.

**Other limitation*Other limitation is*** *is*the lack of investigation of genetic and immunological the lack of investigation of genetic and immunological factors that may contribute to susceptibility to PSE in IgAV patients.

In conclusion, PSE affected approximately one-third of IgAV patients at diagnosis, mainly appearing in the lower and upper limbs. Multiple locations were evidenced in approximately 40 % of them. This skin manifestation was predominantly identified at an early age, with a more inflammatory presentation at onset. However, in spite of the higher levels of inflammatory markers, PSE was not linked to more severe outcomes.

## Ethical approval and consent

The Institutional Ethical Board of HCFMUSP (Comissão de Ética para Análise de Projetos de Pesquisa [CAPPesq]) approved the study (number 70,992,423.8.1001.0068), as well as the other participating centers.

## Data availability

The pertinent data were included in the article, and the raw data will be provided to interested researchers when required.

## Authors’ contributions

Study design and planning: LVS, CRD, MSF, PSM, SDC, LFCF, GC RNM, LMC, FRS, CAAS. Data collection, analysis and interpretation: L.V.S., C.R.D., M.S.F., P.S.M., S.D.C., L.F.C.F., R.N.M., S.C.L.F., G.C., V.C., C.A.L., L.M.C., F.H.R.G., V.P.L.F., R.G.A., F.R.S., L.M.A.C., A.M.E.S., V.A.B., N.E.A., B.O.L.C., M.C.S., K.K., C.A.A.S. Manuscript drafting: L.V.S., C.R.D., M.S.F., P.S.M., S.D.C., L.F.C.F., R.N.M., S.C.L.F., G.C., V.C., C.A.L., L.M.C., F.H.R.G., V.P.L.F., R.G.A., F.R.S., L.M.A.C., A.M.E.S., V.A.B., N.E.A., B.O.L.C., M.C.S., K.K., C.A.A.S. Manuscript review: L.V.S., C.R.D., M.S.F., P.S.M., S.D.C., L.F.C.F., R.N.M., S.C.L.F., G.C., V.C., C.A.L., L.M.C., F.H.R.G., V.P.L.F., R.G.A., F.R.S., L.M.A.C., A.M.E.S., V.A.B., N.E.A., B.O.L.C., M.C.S., K.K., C.A.A.S. Approval of the final version: L.V.S., C.R.D., M.S.F., P.S.M., S.D.C., L.F.C.F., R.N.M., S.C.L.F., G.C., V.C., C.A.L., L.M.C., F.H.R.G., V.P.L.F., R.G.A., F.R.S., L.M.A.C., A.M.E.S., V.A.B., N.E.A., B.O.L.C., M.C.S., K.K., C.A.A.S. Public responsibility for the content of the article: L.V.S., C.R.D., M.S.F., P.S.M., S.D.C., L.F.C.F., R.N.M., S.C.L.F., G.C., V.C., C.A.L., L.M.C., F.H.R.G., V.P.L.F., R.G.A., F.R.S., L.M.A.C., A.M.E.S., V.A.B., N.E.A., B.O.L.C., M.C.S., K.K., C.A.A.S.

## Funding

This study was supported by grants from Fundação de Amparo à Pesquisa do Estado de São Paulo (FAPESP) (#2022/13,837–5 to KK & MC-S) and (#2022/12,925–8 to NEA & CAS) and from Conselho Nacional de Desenvolvimento Científico e Tecnológico (CNPq) (#304,984/2020–5 to CAS).

## Data availability statement

The datasets generated and/or analyzed during the current study are available from the corresponding author upon reasonable request.

## Conflicts of interest

The authors declare no conflicts of interest.

## References

[1] Buscatti I.M., Casella B.B., Aikawa N.E., Watanabe A., Farhat S.C.L., Campos L.M.A. (2018). Henoch-Schönlein purpura nephritis: initial risk factors and outcomes in a Latin American tertiary center. Clin Rheumatol.

[2] Buscatti I.M., Abrão H.M., Kozu K., Marques V.L.S., Gomes R.C., Sallum A.M.E. (2018). Characterization of scrotal involvement in children and adolescents with IgA vasculitis. Adv Rheumatol.

[3] Nussinovitch M., Prais D., Finkelstein Y., Varsano I. (1998). Cutaneous manifestations of Henoch-Schönlein purpura in young children. Pediatr Dermatol.

[4] Júnior C.R., Yamaguti R., Ribeiro A.M., Melo B.A., Campos L.A., Silva C.A. (2008). Lesões vésico-bolhosas hemorrágicas na púrpura de Henoch-Schönlein e revisão da literatura. Acta Reumatol Port.

[5] Sestan M., Kifer N., Sozeri B., Demir F., Ulu K., Silva C.A. (2023). Clinical features, treatment and outcome of pediatric patients with severe cutaneous manifestations in IgA vasculitis: multicenter international study. Semin Arthritis Rheum.

[6] Bouchard M., Sidlow R. (2020). Henoch-Schönlein purpura presenting with subcutaneous edema. J Clin Rheumatol.

[7] Arunath V., Athapathu A.S., Hoole T.J., Aruppala H., Rathnasri A., Ranawaka R. (2020). Severe disfiguring scalp and facial oedema due to Henoch-Schönlein purpura in a child. Case Rep Pediatr.

[8] Kiss M.H., de Sá E.G., Lotufo S.A., Sogabe T., Moretto P.A. (1994). Aspectos clínicos, laboratoriais e terapêuticos de 46 crianças com púrpura de Henoch-Schönlein. J Pediatr (Rio J).

[9] Trapani S., Micheli A., Grisolia F., Resti M., Chiappini E., Falcini F. (2005). Henoch-Schönlein purpura in childhood: epidemiological and clinical analysis of 150 cases over a 5-year period and review of literature. Semin Arthritis Rheum.

[10] Anil M., Aksu N., Kara O.D., Bal A., Anil A.B., Yavaşcan O. (2009). Henoch-Schönlein purpura in children from western Turkey: a retrospective analysis of 430 cases. Turk J Pediatr.

[11] Demircioğlu K., Kiliç B., Kasap Demir B. (2018). Determination of risk factors in children diagnosed with Henoch-Schönlein purpura. Arch Rheumatol.

[12] Karadağ Ş.G., Tanatar A., Sönmez H.E., Çakmak F., Kıyak A., Yavuz S. (2019). The clinical spectrum of Henoch-Schönlein purpura in children: a single-center study. Clin Rheumatol.

[13] Ekinci R.M.K., Balci S., Melek E., Karabay Bayazit A., Dogruel D., Altintas D.U. (2020). Clinical manifestations and outcomes of 420 children with Henoch-Schönlein purpura from a single referral center from Turkey: a three-year experience. Mod Rheumatol.

[14] Basu K., Addya S., Mukherjee S., Sengupta M., Pandey R., Chatterjee G. (2022). Clinicopathological spectrum of Henoch-Schönlein purpura vasculitis: an experience from a tertiary care center. Saudi J Kidney Dis Transpl.

[15] Marcia M., Parodi E. (2022). Lumbar swelling and migrating edema in 3- and 4-year-old boys. SAGE Open Med Case Rep.

[16] Hung T.Y., Liu M.C., Hsu C.F., Lin Y.C. (2009). Facial edema as the initial presentation of Henoch-Schönlein purpura in a 5-year-old boy. Pediatr Emerg Care.

[17] Schaefer B., Soprano C. (2014). Five-year-old boy presenting with severe back pain, swelling, and refusal to walk. Clin Pediatr (Phila).

[18] Duman M.A., Duru N.S., Çalışkan B., Sandıkçı H., Çengel F. (2016). Lumbar swelling as the unusual presentation of Henoch-Schönlein purpura in a child. Balkan Med J.

[19] Yasumura T., Katsumori T., Kizu O., Umehara H., Nakai Y. (2021). Posterior lumbar subcutaneous edema as the rare sign of IgA vasculitis (Henoch-Schönlein purpura): a case of a child. Radiol Case Rep.

[20] Shimizu A., Shimabukuro M., Shimizu M., Asai S., Tomizawa S., Hatakeyama S. (2019). Painful subcutaneous edema of the lumbar region in IgA vasculitis. Pediatr Int.

[21] Ozen S., Pistorio A., Iusan S.M., Bakkaloglu A., Herlin T., Brik R. (2010). Paediatric Rheumatology International Trials Organisation (PRINTO). EULAR/PRINTO/PRES criteria for Henoch-Schönlein purpura, childhood polyarteritis nodosa, childhood Wegener granulomatosis and childhood Takayasu arteritis: ankara 2008. Part II: final classification criteria. Ann Rheum Dis.

[22] Bronz G., Gianini J., Passi A.G., Rizzi M., Bergmann M.M., Milani G.P. (2023). Autoimmune markers and vascular immune deposits in Finkelstein-Seidlmayer vasculitis: systematic literature review. J Autoimmun.

[23] Roman C., Dima B., Muyshont L., Schurmans T., Gilliaux O. (2019). Indications and efficiency of dapsone in IgA vasculitis (Henoch-Schönlein purpura): case series and a review of the literature. Eur J Pediatr.

[24] de Almeida J.L., Campos L.M., Paim L.B., Leone C., Koch V.H., Silva C.A. (2007). Renal involvement in Henoch-Schönlein purpura: a multivariate analysis of initial prognostic factors. J Pediatr (Rio J).

[25] Flynn J.T., Kaelber D.C., Baker-Smith C.M., Blowey D., Carroll A.E., Daniels S.R. (2017). Subcommittee on screening and management of high blood pressure in children. Clinical practice guideline for screening and management of high blood pressure in children and adolescents. Pediatrics.

[26] Moyer V.A. (2013). U.S. Preventive Services Task Force. Screening for primary hypertension in children and adolescents: U.S. Preventive Services Task Force recommendation statement. Ann Intern Med.

[27] Chan J.C., Williams D.M., Roth K.S. (2002). Kidney failure in infants and children. Pediatr Rev.

[28] Levey A.S., Eckardt K.U., Dorman N.M., Christiansen S.L., Hoorn E.J., Ingelfinger J.R. (2020). Nomenclature for kidney function and disease: report of a Kidney Disease: improving Global Outcomes (KDIGO) consensus conference. Kidney Int.

[29] Stevens P.E., Levin A., Disease Kidney (2013). Improving Global Outcomes (KDIGO) CKD Work Group. Evaluation and management of chronic kidney disease: synopsis of the Kidney Disease: improving Global Outcomes 2012 clinical practice guideline. Ann Intern Med.

[30] Cattran D.C., Coppo R., Cook H.T., Feehally J., Roberts I.S.D., Troyanov S., Working Group of the International IgA Nephropathy Network and the Renal Pathology Society (2009). The Oxford classification of IgA nephropathy: rationale, clinicopathological correlations, and classification. Kidney Int.

[31] Modi S., Rajpurohit N., Kumar A. (2016). Acute scrotal involvement in Henoch-Schönlein purpura: a diagnostic dilemma. Urology.

[32] Ha T.S., Lee J.S. (2007). Scrotal involvement in childhood Henoch-Schönlein purpura. Acta Paediatr.

[33] Di Pietro G.M., Castellazzi M.L., Mastrangelo A., Montini G., Marchisio P., Tagliabue C. (2019). Henoch-Schönlein purpura in children: not only kidney but also lung. Pediatr Rheumatol Online J.

[34] Hédin E., Bijelić V., Barrowman N., Geier P. (2022). Furosemide and albumin for the treatment of nephrotic edema: a systematic review. Pediatr Nephrol.

[35] Ozturk K., Cakan M. (2020). Initial manifestations and short-term follow-up results of Henoch-Schönlein purpura in children: a report from two centers. North Clin Istanb.

